# Two cases with discrepancy in the quantitative cytological assessment of cerebrospinal fluid in neonatal samples using light microscopy in comparison with Sysmex XN-1000

**DOI:** 10.11613/BM.2024.020802

**Published:** 2024-04-15

**Authors:** Pavel Broz, Simona Kukralova, Jana Palatova, Klara Kolduskova, Jana Zenkova, Daniel Rajdl, Jaroslav Racek

**Affiliations:** 1 Institute of Clinical Biochemistry and Haematology, University Hospital in Pilsen, Pilsen, Czech Republic; 2 Faculty of Medicine in Pilsen, Charles University in Prague, Pilsen, Czech Republic

**Keywords:** biochemistry, cerebrospinal fluid, cytology, hematology, neonatology

## Abstract

We present two cases from the neonatal department with cerebrospinal fluid examination. We revealed a striking discrepancy in polymorphonuclear (PMN) and mononuclear (MN) cell counts using conventional light microscopy in comparison with automated analyzer Sysmex XN-1000 (PMNs - 13 *vs*. 173x10^6^/L, MNs - 200 *vs*. 67x10^6^/L in case 1 and PMNs - 13 *vs*. 372x10^6^/L, MNs - 411 *vs*. 179x10^6^/L in case 2). We revealed the dominant presence of hemosiderophages in both cases in cytospin slide. Even though Sysmex XN-1000 offers fast examination with a low sample volume, there is possibility of misdiagnosis, with negative impact on the patient.

## Graphical abstract

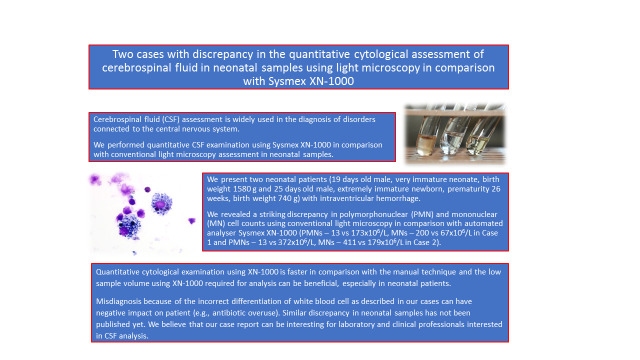

## Highlights

• The low required sample volume for analysis using Sysmex XN-1000 can be beneficial, especially in neonatal patients

• The misdiagnosis because of the incorrect differentiation of white blood cells can have negative impact in cases when the sample volume is insufficient and the cytospin slide is not investigated simultaneously

## Introduction

Cerebrospinal fluid (CSF) assessment is widely used in the diagnosis of disorders connected to the central nervous system. Quantitative cytology is a crucial part of the cytological assessment (*1*). Fast analysis is important in the neonatal population because of the higher incidence of purulent meningitis than at any other age (*2*). Studies have focused on the comparison of routinely performed quantitative CSF cytological analysis using light microscopy and quantitative analysis using hematology analyzer Sysmex XN-1000, with promising results (*3*). We present two cases of clinically interesting results where discrepant results were observed in the quantitative cytological assessment using light microscopy compared with Sysmex XN-1000.

## Laboratory analyses

We performed the total leukocyte count in presented samples from the neonatal department using conventional light microscopy in a Fuchs-Rosenthal (F-R) counting chamber using Samson’s solution. Samson’s solution is the cell counting stain, the main components are glacial acetic acid and magenta dye; the composition allows red blood cells (RBC) fusion and mononuclear (MN) and polymorphonuclear (PMN) cells including differentiation. We prepared eosin-thiasin-stained slides using the cytospin technique and examined them under the light microscope. The differentiation of MN and PMN cells was assessed subsequently. A native CSF sample was simultaneously analyzed on a hematological analyzer Sysmex XN-1000 (Sysmex, Cobe, Japan) using body fluid (BF) mode. The Sysmex XN-1000 is a stand-alone analyzer that uses a combination of electrical impedance, laser light scattering and dye binding for measurement. The analyzer is normally used to count elements from whole blood in whole blood mode. However, the Sysmex XN-1000 can be used in BF mode to measure other body fluids other than whole blood. The work was approved by the Ethics Committee of the University Hospital and Medical Faculty in Pilsen, reference number 62/23. Written informed consent was obtained from the patient’s relatives for publication in a medical journal.

## Case presentations

Case 1 is a 19 days old patient, initially very immature neonate, prematurity 30 weeks, birth weight 1580 g. Due to intraventricular hemorrhage grade III bilaterally and posthemorrhagic hydrocephalus, he was admitted to the neonatal intensive care unit. He underwent neurosurgical procedure with placement of subgaleal ventricular reservoir.

Case 2 is a 25 days old patient, extremely immature newborn, prematurity 26 weeks, birth weight 740 g. Due to intraventricular hemorrhage grade III (left) and IV (right) and posthemorrhagic hydrocephalus, he underwent neurosurgical procedure with subgaleal ventricular reservoir placement.

**Figure 1:**
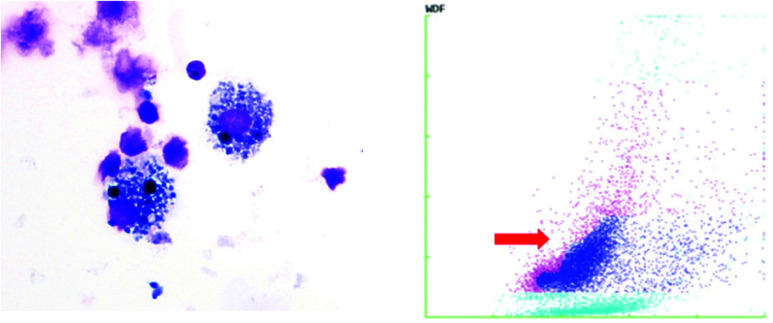
Dominant presence of hemosiderophages on the cytospin slide in case 2 (May-Grunwald-Giemsa, magnification 400x) (A). Scattergram of cerebrospinal fluid sample in case 2. Merging scattergram represent atypical cluster of polymorphonuclear (dark blue) and mononuclear (pink) cells (B). X-axis - nucleic acid content, Y-axis - internal complexity.

Repeated CSF analyses were performed in both cases. The results of CSF analyses with clearly discrepant results of quantitative cytological examination of CSF by light microscopy and Sysmex XN-1000 are presented in Table 1. We examined stained slides from both patients and revealed the dominant presence of hemosiderophages (Figure 1A). A scattergram showing atypical cluster of PMN and MN cells is presented in Figure 1B.

**Table 1:** Presentation of case 1 and case 2 with discrepant results using a Fuchs-Rosenthal counting chamber and Sysmex XN-1000

		**WBC (x10^6^/L)**		**PMN (x10^6^/L)**		**MN (x10^6^/L)**	
**Case**	**Age (days)**	**F-R**	**Sysmex** **XN-1000**	**F-R**	**Sysmex** **XN-1000**	**F-R**	**Sysmex** **XN-1000**
1	19	213	240	13	173	200	67
2	25	424	551	13	372	411	179
WBC - white blood cells. PMN - polymorphonuclear cells. MN - mononuclear cells. F-R - Fuchs-Rosenthal.							

## Discussion

Even though some authors describe a possible discrepancy in CSF samples with regard to the presence of macrophages, mesothelial cells or tumor cells, a similar discrepancy in neonatal samples with the dominance of hemosiderophages has not been published yet (*4*-*6*). Siderophages are macrophages containing vacuolated cytoplasm with bluish hemosiderin pigment as a result of hemoglobin degradation. Their presence is usually associated with bleeding into the subarachnoid space during subarachnoid hemorrhage or may be present in patients following neurosurgery. Single hemosiderophages may be present after repeated lumbar punctures (*1*). Experienced laboratory specialist is able to count hemosiderophages in F-R counting chamber as MNs due to their non-lobulated nucleus compared to PMNs. In addition, the presence of hemosiderophages as MNs can be easily confirmed in stained slide due to their characteristic appearance.

Fleming speculated whether discrepancy in cell count in CSF samples can be caused by the presence of cell debris, the loss of cells during centrifugation in cytospin slide preparation or by the fact that the cell number decreases rapidly as a result of cell lysis (*7*). However, even though some of these possibilities can be taken into account, the striking discrepancy as that in our case cannot be explained. Fleming and colleagues report that the samples in their study were analyzed using analyzer *prior* to manual analysis (*7*). However, in our presented cases, samples were counted manually and subsequently analyzed using Sysmex XN-1000. The total leukocyte count was higher using Sysmex XN-1000 as presented in Table 1. Lysis caused by the cytospin technique cannot explain the discrepancy in our samples.

High-fluorescence cells, such as macrophages, mesothelial cells or tumor cells, can be incorrectly counted in the scattergram or can be excluded from the white blood cell (WBC) count, potentially generating the error „WBC abnormal scattergram“ (*7*, *8*). Although some authors recommend using the protocols in case of such errors, according to our observations, this flag is not always generated when highly discrepant results are present (although we observed similar errors in our laboratory, we present only samples without this flag) (*6*). According to our experience, laboratory specialists performing CSF assessments using an automated analyzer have to be experienced in order to recognize possible abnormalities in scattergrams or to investigate cytological slides.

Although Sysmex XN-1000 offers satisfying results with limit of quantification (LoQ) 5x10^6^/L, using the body fluid mode, some authors reported that the performance of analyzers in comparison with the manual method in samples with low cellularity does not have to be sufficient, as in cases where the RBC is relatively high and the WBC is low (*3*-*5*). However, according to our experience, the prevalence of bloody CSF samples in neonatal samples is higher in comparison with other populations. A possible solution could be to perform the analysis using an automated analyzer only in macroscopically “turbid” samples, which are not macroscopically clotted or extremely bloody.

The sample volume from neonates is generally lower than from other patients. The advantage of Sysmex XN-1000 is the lower sample volume used for analysis (88 μL), however 160 μL is required for analysis. The necessary volume for assessment using the counting chamber is 100 µL, with an additional 50-400 µL for cytospin slide assessment according to the RBC and WBC count. Thus, we struggle with an inadequate sample volume in a substantial percentage of neonatal patients and a fast and reliable cytological assessment using low sample volumes would be beneficial especially in these cases.

Quantitative cytological examination using Sysmex XN-1000 is faster in comparison with the manual technique and the low sample volume required for analysis can be beneficial, especially regarding neonatal patients. However, the misdiagnosis because of the incorrect differentiation of WBC can have negative impacts (*e.g.*, antibiotic overuse), especially in cases when the sample volume is insufficient and the cytospin slide is not investigated simultaneously. We believe that future software updates of similar analyzers might also eliminate this problem.

In conclusion, we observed a striking discrepancy in quantitative CSF examination using Sysmex XN-1000 in comparison with conventional light microscopy assessment in neonatal samples where hemosiderophages are dominant. Although Sysmex XN-1000 offers a rapid examination with a small sample volume, cytospin slides should be evaluated simultaneously because of the possibility of misdiagnosis if cytological evaluation of is not performed. Thus, laboratory specialists performing CSF assessments using an automated analyzer have to be experienced in order to recognize possible abnormalities in scattergrams or to investigate cytological slides.

## Data Availability

All data generated and analyzed in the presented study are included in this published article.
